# Gamma oscillations as a biomarker for major depression: an emerging topic

**DOI:** 10.1038/s41398-018-0239-y

**Published:** 2018-09-04

**Authors:** Paul J. Fitzgerald, Brendon O. Watson

**Affiliations:** Department of Psychiatry, University of Michigan, Ann Arbor, MI 48109-5720 USA

## Abstract

Identifying biomarkers for major depression is of high importance for improving diagnosis and treatment of this common and debilitating neuropsychiatric disorder, as the field seeks to move toward both personalized and more effective treatments. Here we focus on electroencephalography (EEG) or direct scalp voltage recordings as such a biomarker, with an emphasis on gamma and high gamma oscillations (or “rhythms”). In the last several decades, alpha and theta band rhythms have been found to provide information on depressive state as well as recovery, but the gamma band is less well characterized with respect to depression. We summarize some key findings on gamma rhythms (especially their amplitude) as a biomarker or endophenotype for major depression. These studies suggest: (1) under certain conditions gamma rhythms can distinguish subjects with major depression from healthy controls, (2) gamma may distinguish bipolar disorder from unipolar depression, (3) various pharmacological and non-pharmacological treatments that counteract depression also alter gamma, (4) animal models of depression-like behavior show gamma abnormalities, with changes in gamma associated with therapeutic recovery. The most informative approaches in the future may combine profiles of gamma band power across the brain to assess ratios of activity across regions. Overall we have good evidence to suggest that gamma rhythms may provide objective information on major depressive disease status, but we will need further work to better define the precise measures to follow.

## Introduction

A major challenge in implementing precision medicine approaches in psychiatry is to identify sensitive and reliable biomarkers for major depression. Doing so would be of critical import for improving diagnosis and treatment of this prevalent and debilitating neuropsychiatric disorder. In this publication, we focus on electroencephalography (EEG) or other voltage recordings as such a candidate biomarker, with an emphasis on gamma oscillations (or “rhythms”). EEG, along with invasive local field potential (LFP) recordings, has the ability to reveal voltage events and oscillations emanating from and within the brain in characteristic frequency bands, which have been given names such as theta (4–8 Hz) and alpha (8–12 Hz), gamma (30–100 Hz) and sometimes high gamma/high-frequency oscillations (HFO; 100–200 Hz). Here we focus on the gamma band and define it broadly to include all oscillations 30–200 Hz, including what are sometimes called HFO. This choice is based firstly on the spectral heterogeneity reported in the literature, with often partially but incompletely overlapping frequency bands defined as gamma, and secondly on neurophysiologic evidence that the 50–200 Hz band has a monotonic and uniform relationship with firing rates of local neurons^1–4^ and may share common mechanisms^5,6^. Despite the spectral and anatomical inconsistencies between the studies, we believe based on the evidence above that the gamma oscillation represents a sufficiently unified frequency band, such that it is of interest to review the current findings with an eye towards possible future implications despite some degree of inconsistency. In the end, we will make recommendations regarding the irregularities and conflicts in the current literature.

In the last several decades, alpha and theta band rhythms have been found to provide information on depressive state as well as recovery^7–9^, but the gamma band is less well characterized—despite our emerging understanding of its importance for neural processing in both humans and rodents^10^. Here we summarize some key findings regarding gamma rhythms (especially their “power”, i.e., amplitude) as a biomarker or endophenotype for major depression. These studies suggest: (1) under certain conditions gamma rhythms can distinguish subjects with major depression from healthy controls, (2) gamma may distinguish bipolar disorder from unipolar depression, (3) various pharmacological and non-pharmacological treatments that counteract depression also alter gamma, (4) animal models of depression-like behavior show gamma abnormalities, with changes in gamma associated with therapeutic recovery. These lines of evidence collectively suggest that gamma rhythms may provide objective information on major depressive disease status. In this review we provide more detail and analysis of these issues, with an eye toward the future of this emerging topic.

## Gamma rhythms: background information

Before reviewing the human literature it is worthwhile to briefly discuss advances in our understanding of gamma rhythms largely from rodent experimental work, where new tools have allowed us to make significant advances. EEG rhythms are broadly believed to be important in sculpting spike-timing and cooperative coding across brain regions^11^ and are prominent in many brain regions. Gamma rhythms indeed correlate with increased neuronal action potential generation^4,12^, including when sensory stimuli are received and processed^13,14^. Similarly, hippocampal gamma oscillations are strongly correlated with times of information encoding^15,16^, and gamma range rhythms may facilitate hippocampo-cortical coordination during cognitive tasks^17^.

## Gamma rhythms in depressive disorders

A number of studies suggest that gamma rhythms differ between healthy controls and individuals with unipolar depression. The experimental approaches used in these studies vary, with some measuring gamma power during cognitive tasks^18,19^, and others analyzing the oscillatory power spectrum at rest (i.e., baseline)^20–22^. These studies have used various gamma frequency sub-bands, anatomical regions and behavioral states ranging from memory or emotional tasks, to sensory-evoked measures, and not surprisingly have yielded varying results. For example, an EEG study found that subjects with high depression scores (including Beck Depression Inventory (BDI) and Mood and Anxiety Symptom Questionnaire (MASQ) scores) had reduced resting gamma in the anterior cingulate cortex^22^, whereas gamma increased in frontal and temporal regions in a study in which subjects with depression performed spatial and arithmetic tasks^19^. In addition, subjects performing emotion-related tasks in major depression can show decreased frontal cortex gamma^23,24^. Akar et al.^20^ found increased resting complexity of gamma signaling in frontal and parietal cortex, in subjects with major depression. Given the task- and anatomy-specific nature of gamma band activity shown in rodent work, it is highly likely that this heterogeneity in the human literature reflects the varying conditions used in different studies. Future studies will need to further address the task-specific or sensory-based approaches for modulating gamma, as these various experimental manipulations may not reflect the same underlying physiological mechanisms. Nonetheless, it appears that gamma rhythms, under some of the conditions described above, do provide information regarding depressive status.

A number of studies also suggest that gamma rhythms in individuals with unipolar depression are distinct from those found in bipolar disorder, including bipolar depression. These studies report differences between the two clinical populations in a brain region-specific manner, with both increases and decreases in gamma power in different regions, depending on the disorder and task used. Some of these regional differences include frontal, parietal, and temporal cortex^23–27^. For example, a magnetoencephalography (MEG) study that used auditory-evoked stimulation found that subjects with unipolar depression had greater gamma power brain-wide than those with bipolar disorder^25^, and likewise a group of individuals with bipolar disorder showed decreased auditory-evoked gamma in a variety of brain regions relative to healthy controls^27^. In comparing subjects with bipolar disorder versus unipolar depression during emotional tasks, two of these studies found increases in gamma power in temporal regions in unipolar depression^23,26^, whereas two of these studies found decreases in frontal gamma power in this disorder^23,24^. Collectively, these five studies suggest that both emotional and sensory tasks or stimuli can distinguish unipolar from bipolar depression.

## Action of depression treatments on gamma rhythms

Pharmacological studies give us further insight into the role of gamma in major depression—especially studies using medications that have the ability to reverse depressive symptoms. Overall, these studies reveal relationships between gamma signaling and particular antidepressant drugs, or classes of drugs, and possibly their underlying signaling pathways. Serotonergic and noradrenergic drugs, for example, have opposing effects on gamma power in various brain regions. The serotonin boosting antidepressants, citalopram and fluoxetine, suppress gamma in rats^28,29^ and similarly, evoked serotonin release through electrical stimulation of the dorsal raphe nucleus in animals also decreases gamma power^30^. In contrast, systemic administration of the noradrenergic antidepressant, reboxetine, increases gamma power, with fairly similar overall effects to the noradrenergic tricyclic antidepressant, desipramine, in this study^31^. These opposing effects on gamma power produced by serotonergic versus noradrenergic antidepressants, do not contradict the hypothesis gamma oscillations are a biomarker for major depression. Instead, since some individuals with depression respond to one type of drug and not the other, perhaps depression is characterized by elevated versus decreased gamma signaling in particular circuits in a given case^32^. In this scenario, an “optimal” amount of gamma may indicate euthymia, where serotonergic versus noradrenergic drugs could be used in such cases to implement precision medicine.

Treatment with antidepressant-dose ketamine brings with it an important new perspective on gamma oscillations in depression. The observation that ketamine is not only efficacious in helping a large proportion of patients with depression, but that it can do so in a manner that is both rapid and long-lasting implies it may have a fundamentally different mechanism of action from other antidepressants^33^. Therefore an understanding of the mechanism of action of ketamine as an antidepressant may yield novel insights into depressive circuitry. In fact the most well characterized and perhaps most prominent electrophysiologic effect of ketamine is an increase in gamma rhythm power. In rodents, acute systemic administration (and in some cases, local brain infusion) of ketamine enhances gamma power in a variety of brain regions^34,35^. This occurs early after administration and precedes lasting mood effects. Human studies also show this prominent boost of gamma power immediately post-administration and therefore preceding most mood effects, possibly implying it could play a role in the mechanism of action of this medication^36–38^. A recent MEG study that compared ketamine response in depression with that in healthy controls found that this drug had an antidepressant effect in the overall population of individuals with depression, while inducing mild depression in the controls^32^. This study also found that higher drug-induced gamma power across multiple brain regions was associated with better response in subjects with depression who had lower baseline gamma, whereas individuals with higher baseline gamma had a poorer response. Overall, this study suggests that an “optimal” amount of gamma power in diverse circuits may be associated with euthymia (see below). An important unresolved question here is whether gamma oscillations are simply a marker of drug action versus being a causative mediator of drug effect. We suggest here that gamma is actually causal with respect to the therapeutic actions of ketamine, as well as monoaminergic antidepressants.

Similarly, non-pharmacologic treatment for depression using transcranial magnetic stimulation (TMS) also points to gamma rhythms as key indicators of treatment success. A number of studies find increases in (particularly resting) gamma signaling after recovery from depression^39–41^. Noda et al.^39^ showed, for example, that therapeutic recovery from major depression is associated with increases in prefrontal gamma power, as well as measures of theta–gamma coupling.

Bipolar depression on the other hand provides a less straightforward picture. Canali et al.^42^ found that after recovery from bipolar, rather than unipolar, depressive episodes TMS-evoked gamma power remains reduced. The authors suggest that gamma rhythm changes may be more trait-than-state related in patients with bipolar disorder^42^. A possible confound in this study is the elevated likelihood of manic, hypomanic, or subclinical manic-like episodes after recovery from bipolar depression. An additional TMS study of bipolar depression found that recovery is associated with a decrease in baseline gamma^43^. Likewise, deep brain stimulation in treatment-resistant depression can suppress task-evoked gamma in association with reduced symptomatology^44^, similar to results from mindfulness-based cognitive therapy in recurrent depression^45^.

## Animal studies of depressive-like states

Gamma rhythms in depression-related behavior are an active subject of animal research as well. Rodent models show that gamma rhythms are altered in depression-related states, and that treatments in rodents induce changes in gamma during therapeutic recovery^46,47^. Two rodent models displaying depression-like behavioral phenotypes, Flinders sensitive line (FSL) rats and mice expressing the truncated Disrupted-in-schizophrenia 1 (Disc1) mutation, show deficits in gamma signaling^48,49^. These and future animal studies complement the human subject work on gamma in depression, where linkages of these rhythms with mood circuitry represents a promising future direction since it allows for invasive techniques that are not feasible in clinical research.

Additional animal studies show that changes in the gamma frequency band are strongly associated with changes in neural inhibitory signaling and specifically may relate to altered excitatory/inhibitory (E/I) balance or ratio, which has recently been implicated in major depression^50^. Fee et al.^50^ also reviewed postmortem brain tissue data and proton magnetic resonance spectroscopy evidence in living individuals suggesting inhibitory interneuron dysfunction and low GABA levels in subjects with depression. This dysfunctional signaling was present in a variety of brain regions, including prefrontal cortex, amygdala, and anterior cingulate cortex. LFP recordings have recently suggested that in rats, gamma power and E/I ratio are inversely related across a variety of waking and sleeping states^4^. Changes in gamma associated with E/I balance may also be accompanied by long-term alterations in dendritic plasticity, particularly with respect to somatostatin-positive GABAergic interneurons^50^. Whereas the circuit mechanisms that produce gamma oscillations under these circumstances are not completely understood, recurrently coupled networks of interneurons may play a role^51^.

## Technical limitations and recommendations

Based on the data presented here, gamma rhythms appear to correlate with depressive status and in fact may correlate with therapeutic response. This opens the possibility of using this brain rhythm as a biomarker and may also lead us to improved therapeutics via either individualized treatment or alteration of gamma rhythms themselves, should they prove causal in either depression or therapeutic response to depression.

Frequency band and anatomical location represent two major dimensions of variance in the literature. With regard to frequency band defining “gamma”, many studies use different frequency ranges to define “gamma”, so greater standardization would facilitate further work. We would specifically suggest that future work should avoid defining idiosyncratic frequency bands in each study, which leads to both heterogeneity and a low-resolution picture of the phenomenon. Rather we recommend that future studies apply analyses to all recordable frequency bands, which will lend itself to cross-study comparison and yield greater information as well. Specifically, using dozens of logarithmically-spaced frequency bins spanning from 1–200 Hz is a pragmatic suggestion given that it does not assume that the experimenter can determine the band of interest prior to the study^4^. With modern computers such analyses are not difficult and we suggest them as a new default.

With regard to anatomical location, it is clear that the field will need better information regarding which brain regions are most critical in depression. Given that gamma oscillations often correlate with spiking activity level, relative gamma band power fluctuations may essentially define relative activity levels and balances between cooperating or offsetting brain regions. Therefore similar to our recommendation that researchers report results from a full span of frequency bands, we recommend that researchers provide data regarding the full anatomical distribution of their electrodes.

Additionally, brain state may matter and not all studies are done under the same state. Since gamma and other bands are modulated across sleep-wake and other brain states^52^, gamma should be considered in the context of the brain state of the subjects. For instance, inactive and possibly drowsy subjects may show very different modulation compared to subjects engaged in active tasks. We therefore suggest that at the least, future studies specify brain state and even better they might report findings across brain states. Furthermore sleeping patterns are typically disturbed during major depression, and so studying the relationship between sleep and gamma is a promising avenue for future inquiry^53^. Furthermore, therapeutic changes in gamma signaling may affect brain regions comprising the default mode network, whose functional activation is enhanced during major depression, and in this network gamma rhythms are known to be suppressed when an individual carries out an external, attention-demanding task^54^.

A variety of approaches may be applied going forward to verify and further elucidate the role of gamma brain rhythms in depression and other psychiatric disorders. These include both invasive and non-invasive electrophysiology, TMS, MEG, and functional brain imaging^55^. Regarding fMRI in particular: whereas the blood oxygenation level dependent (BOLD) signal can co-localize with gamma modulation^56^, this is not always the case^57^, suggesting that EEG gamma measures may provide additional information as a candidate biomarker. One possibility is that since resting (i.e., baseline) gamma may be easier to measure than task- or sensory-induced paradigms, which can require additional equipment or training of subjects, resting gamma in particular may be pragmatic as a biomarker for major depression once properly defined spectrally and anatomically. Another consideration is that in subjects with depression, EEG neurofeedback^58^, if effective, might be used to modulate and monitor gamma power, while simultaneously inducing therapeutic cognitive or affective changes in the individual.

## Outlook: additional concepts and considerations

Several studies cited above implicate gamma rhythms as a state-related biomarker rather than a trait-related one (or endophenotype)—in that gamma correlates with depressive status at a given point in time. This may be particularly clear in the case of unipolar depression; however, at least some work suggests that gamma power is an indicator of trait in the case of bipolar disorder^42^. Future studies could further address gamma as a state versus trait marker in bipolar disorder, by comparing these rhythms during mania or hypomania versus depression in the same individual. If gamma is a state-related biomarker for mood, it could be used for diagnosis and in principle also be used to monitor a variety of ongoing treatments, both pharmacological and non-pharmacological, for these disorders. If gamma is actually more trait-related, it could still be used to more effectively diagnose depression.

Further testing of the gamma-biomarker hypothesis will also include how gamma rhythms may interact with other candidate biomarkers for depression, including other frequency bands^20,21,59^. Human EEG studies in the past several decades have shown that lower frequency bands, such as theta and alpha, are also promising candidate biomarkers^7–9^. In this regard, combining gamma spectral information with that from other frequency bands, including cross-frequency coupling, may be superior to using only one in isolation^40^. The findings of a particularly powerful gamma rhythm after administration of the uniquely acting agent ketamine and prior to frank mood effects points to gamma rhythms as a particularly important target for future exploration.

It should be noted that EEG rhythms have been particularly well studied in the hippocampus and the cortex. In these regions, gamma rhythms likely represent not a single entity but a mixture of both true regular “oscillations” and irregular “rhythms” in the frequency bands above 30 Hz, and these different signaling modes may occur under various tasks or brain states^4,10,12,14,60^. Neuronal spiking activity can also underlie high gamma or HFO, or these oscillations can modulate cortical spiking probability^61^. In the animal literature, the differences between the various types of gamma are only beginning to be observed and understood, and in the human literature, especially the mood-related literature, they have not yet been parsed in most studies. Another consideration is that baseline or resting versus task- or sensory-evoked gamma may invoke distinct types of gamma. It is not clear, in addition, whether EEG scalp recordings versus intracranial or intra-brain recordings may differ—for instance low-pass filtering or volume averaging by tissue may have an effect on EEG recordings. Therefore the information presented on EEG recordings of gamma as a biomarker for depression may relate more directly with cortical signaling rather than deeper, subcortical transmission. Another caveat to using EEG data to monitor gamma is that source localization techniques are often used to infer the brain regions that are generating the EEG patterns, and typically only provide an approximation of their locations^62^. Measuring gamma oscillations with EEG can also present methodological difficulties, due to their high frequency and low amplitude qualities as well as possible physiological artifacts, in contrast with capturing lower frequency bands such as theta and alpha^63^. Collectively, these factors need to be considered as the field continues to investigate the relationship between gamma and major depression.

In this brief review on the emerging topic of gamma as a biomarker for depression, we have not addressed in detail the various aspects of biomarkers, such as strength and specificity, put forth by Bradford Hill^64^. But regarding specificity to a particular neuropsychiatric disease, perhaps the current hypothesis could be criticized because gamma oscillations are known to be perturbed in schizophrenia^65^, as well as in major depression. We suggest here that gamma may provide information on both disorders (as well as bipolar disorder), perhaps due to their overlapping genetics, pathophysiology, and symptomatology (i.e., negative signs in schizophrenia)^66^ and due to the fact that gamma rhythms carry such generalized information about local neural activity. A similar argument can be made for autism spectrum disorder^67^ and epilepsy^68^, including the fact that several of these disorders are often comorbid^69–71^. One possibility is that gamma measured in different brain regions, perhaps especially when combined with additional biomarkers, may help distinguish these various disorders from one another, or may in general help in detecting their comorbidity. Limited data already suggest, for example, that gamma abnormalities are present in prefrontal cortex in both major depression and schizophrenia^19,20,23,24,72^.

Finally, we would also like to emphasize that much of the work to date is descriptive and has not yet developed into causal manipulations to verify the role of gamma rhythms in mood state. Neuroscientists who work with animals therefore have an important opportunity now to apply causal tests, such as optogenetics and chemogenetics, to otherwise observational studies. And translation between animal and human subject work will be critical to building a proper understanding of brain rhythms in depressive disorders.

## Conclusions

In summary, gamma rhythms as a novel biomarker (or “neural readout”) of major depression is an emerging topic, with intriguing results so far, that would benefit from further inquiry through additional controlled studies. There are already sufficient data to make some specific hypotheses but many exciting experiments are yet to be carried out, including identifying changes in gamma signaling in a more brain region-specific manner. For example, since EEG recordings in human subjects may reveal gamma signaling in the cortex more effectively than in subcortical structures, perhaps gamma power in prefrontal cortex in particular may be a reliable marker for major depression, as this brain region is heavily implicated in mood and emotional regulation^73^. These future studies may eventually reveal that optimal mood is associated with an “optimal amount” of gamma signaling^32^, or gamma properly balanced across particular involved brain regions, allowing for effective interregional communication across disparate brain networks, where too much gamma coupling would render local neural spiking too synchronous, and too little would yield disorganized spiking^74^. In this scenario, optimal gamma may reflect a functionally optimal balance between excitation and inhibition in key microcircuits^4,50^, such as those in prefrontal cortex or hippocampus. Combining data on gamma rhythms with that of other frequency bands, as well as other types of candidate biomarkers, could be a fruitful future direction in the quest for personalized medicine in psychiatry. In fact, complex gamma oscillatory patterns across the brain may be more indicative of disease state or treatment response than any simplified metric in any one region. Given that gamma and high gamma rhythms are related to the activity levels of localized brain regions, any focus on these rhythms may need to be task, state, and region specific in order to properly predict disease.
